# A comprehensive approach in high-grade glioma management: position statement from the Neuro-Oncology Scientific Club (NOSC), Shiraz, Iran

**DOI:** 10.3205/000246

**Published:** 2017-02-28

**Authors:** Mansour Ansari, Ahmad Mosalaei, Niloufar Ahmadloo, Alireza Rasekhi, Bita Geramizadeh, Ali Razmkon, Kazem Anvari, Mohammad Afarid, Ali Dadras, Leila Nafarieh, Mohammad Mohammadianpanah, Hamid Nasrolahi, Seyed Hasan Hamedi, Shapour Omidvari, Mohammad Nami

**Affiliations:** 1 Department of Radiation Oncology, School of Medicine, Shiraz University of Medical Sciences, Shiraz, Iran; 2 Department of Radiology, School of Medicine, Shiraz University of Medical Sciences, Shiraz, Iran; 3 Department of Pathology, School of Medicine, Shiraz University of Medical Sciences, Shiraz, Iran; 4 Department of Neurosurgery, School of Medicine, Shiraz University of Medical Sciences, Shiraz, Iran; 5 Cancer Research Center, Mashhad University of Medical Sciences, Mashhad, Iran; 6 Behestan Medical Scientific Committee, Behestan Group, Tehran, Iran; 7 Institute of Biochemistry and Biophysics (IBB), University of Tehran, Tehran, Iran; 8 Department of Neuroscience, School of Advanced Medical Sciences and Technologies, Shiraz University of Medical Sciences, Shiraz, Iran; 9 Neuroscience Laboratory (Brain, Cognition and Behavior), Department of Neuroscience, School of Advanced Medical Sciences and Technologies, Shiraz University of Medical Sciences, Shiraz, Iran; 10 Shiraz Neuroscience Research Center, Shiraz University of Medical Sciences, Shiraz, Iran

**Keywords:** neuro-oncology, interdisciplinary, high-grade glioma, position statement, Shiraz

## Abstract

Establishing a robust teamwork model in the practice of neuro-oncology requires continued interdisciplinary efforts. The Neuro-Oncology Scientific Club (NOSC) initiative is an interdisciplinary clinical forum promoting the comprehensive approach across involved disciplines in the management of central nervous system (CNS) malignancies. With its provincial founding panels and national steering board, NOSC has been operational in Iran since 2011. This initiative has pursued its mission through interval strategic meetings, tumor boards, case discussions as well as publishing neuro-oncology updates, case study periodicals, and newsletters. A provincial meeting of NOSC in Shiraz put together insights from international practice guidelines, emerging evidence, and expert opinions to draw a position statement on high-grade glioma management in adults. The present report summarizes key highlights from the above clinical forum.

## Zusammenfassung

Die Schaffung eines robusten Teamwork-Modells in der Praxis der Neuro-Onkologie erfordert fortgesetzte interdisziplinäre Anstrengungen. Der Neuro-Oncology Scientific Club (NOSC) ist ein interdisziplinäres klinisches Forum, das den umfassenden Ansatz bei der Behandlung von Malignomen des zentralen Nervensystems (ZNS) über alle Fachrichtungen hinweg fördert. Seit 2011 ist NOSC mit seinen regionalen Gründungspanels und dem nationalen Steuerungsgremium im Iran tätig. Diese Initiative hat ihre Mission durch strategische Intervalltreffen, Tumor Boards, Falldiskussionen sowie die Veröffentlichung von Neuroonkologie-Updates, Fallstudienzeitschriften und Rundschreiben verfolgt. Eine regionale Tagung des NOSC in Shiraz brachte Erkenntnisse aus internationalen Praxis-Leitlinien, neue Belege und Expertenmeinungen zusammen, um ein Positionspapier zur Behandlung hochgradiger Gliome bei Erwachsenen zu erstellen. Der vorliegende Bericht fasst die wichtigsten Highlights aus dem oben genannten klinischen Forum zusammen.

## 1 Interdisciplinary care in neuro-oncology: five years with NOSC

The Neuro-Oncology Scientific Club (NOSC) has been an ongoing interdisciplinary initiative fostering teamwork in optimal management of brain tumors across Iran since 2011. Over the past six years and under the NOSC umbrella, experts and practitioners from the disciplines involved in brain tumor care have come together to bridge science, knowledge and practice gaps through research endeavors, educational forums and concurrence on clinical pathways in brain tumor management, respectively [1], [2], [3], [4], [5], [6], [7], [8], [9], [10].

The NOSC comprises provincial and nation-wide steering boards through which its educational and clinical activities are scheduled, planned and implemented. The provincial NOSC founding panel in Shiraz, Southern Iran, hosted an interactive clinical forum entitled: “setting standards for optimal care to high-grade glioma patients through interdisciplinary efforts” where over 50 experts and professionals from related disciplines including neurosurgery, radiation oncology, pathology, neuroradiology, neurology, and clinical neuroscience participated to discuss the most updated evidence.

Discussions during this NOSC forum revolved around:

1. the significance of a comprehensive approach to diagnosis, treatment and follow-up in CNS malignancies,
2. updates on the molecular pathogenesis of glioblastoma multiforme (GBM),
3. advanced imaging technologies in high-grade gliomas,
4. novel surgical approaches in glioma,
5. non-surgical management of high-grade gliomas (HGGs).

The present report highlights the communicated insights during the above NOSC meet-up and summarizes the agreed position by Shiraz-NOSC’s expert panel on “optimizing interdisciplinary care in HGGs”.

## 2 Malignant gliomas and the unsettled clinical burden

The diagnosis, morphological classification, and efficient treatment of HGGs are regarded as challenging clinical encounters. The clinicopathological data are often insufficiently satisfactory to realize the biology of tumors and to timely identify the therapeutic implications as well as the prognosis [11], [12].

The primary glioblastoma multiforme (GBM) predominantly feature astrocytic differentiation while its secondary types may result from astrocytic, oligodendroglial or mixed tumoral transformation of glial cells.

According to the World Health Organization (WHO) classification, GBM is regarded as the most common primary brain tumor with preponderating astrocytic differentiation. GBM is basically identified through atypical, mitotic, and pleomorphic glial cells together with necrosis, vascular thrombosis, micro-vascular proliferation [13], [14]. The more recent WHO update in 2016 has incorporated molecular markers in glioma classification. This classification includes new genetically identified entities and variants, allowing more diagnostic precision, and a new layered approach to diagnosis. It includes newly recognized entities, variants, and patterns, such as IDH (isocitrate dehydrogenase) mutant and wild-type entities. The update also includes designation of new, genetically defined entities such as loss of heterozygosity (LOH) of 1p 19q [15]. The new WHO classification however focuses exclusively on diagnosis. While it includes more molecular assay findings criteria, the specific methods to obtain them are less specified [15].

Promulgating the new classification to our practice requires a balancing act not only to address the needs of both clinicians and patients, but also clinical trial experts, population health researchers, policymakers, and health insurers. A key question to address is “whether in our practice a CNS malignancy is to be defined based on histology and genetics or remains based on histology alone”.

The widespread availability of the new diagnostic technologies needs to be ensured when recommending these tests for diagnosis and classification purposes.

Apart from our country’s major provinces which currently have access to such tests (i.e. IDH-1 mutation test via immunohistochemistry and LOH of 1p 19q test via fluorescence in situ hybridization), institutions or regions lacking access to such diagnostic tools need to be accommodated before the routine use of such test for diagnosis purpose is recommended by our working group. Where such diagnostic tests are not readily accessible, the “not otherwise specified” (NOS) category would be applied when the pathology report is based on the recent WHO classification. 

HGGs comprise a large number of primary brain neoplasms. Though we have started epidemiological surveys using the NOSC brain tumor collaborative registry (BTCR), the prevalence record for HGGs in Iran is yet to be established [3]. In general, GBM is known to account for up to 15% of the intracranial and 60–70% astrocytic tumors, respectively [16], [17], [18].

Similar to a population-based study conducted in Europe which showed that the incidence of GBM peaks at almost the age of 60, and over 80% of the cases were older than 50 years, interim results from our BTCR suggest a comparable trend [6], [18]. In agreement with the above study, our so far records confirm that GBM is more common in males than females [6]. 

Given the infiltrative nature of HGGs and their interference with critically functional and eloquent brain regions, they are typically not amenable to total resection. With a high capacity for micro-infiltration, spreading and rapid progression, survival with GBM tends to be below one year in almost half of the patients [19], [20]. 

Upon confirmation of the primary or secondary GBM diagnosis, the overall survival (OS) is roughly similar. However in secondary GBM, OS depends on the grade of the original pathology which transformed to GBM [21].

When the genetic aspects of the tumors are studied, a large number of mutations may be observed. In many instances, while proto-oncogenes are invigorated, oncogene-protecting factors are suppressed. Studies have suggested a long list for gene mutations both for the primary and secondary GBM [22], [23], [24].

In terms of the GBM location, they may appear in any subcortical region of either hemispheres. While almost in two third of the cases GBMs are found in temporal and parietal lobes, they are observed in frontal and occipital lobes in almost 25% and 16% of the cases, respectively. The fronto-temporal presentation of HGGs and GBM in particular is also frequent [25], [26].

Tumors may infiltrate via the white matter tracts and through the corpus callosum to conquer the contralateral hemisphere. In children, the presence of HGGs in striatum and the thalamic region is not uncommon. Some infrequent or exceptional locations of the tumor include the ventricles, brainstem, cerebellum, and spinal cord [27].

The clinical manifestations of GBM cases may largely vary. In fact, many of the symptoms suggestive for the pathology (including headache, nausea and vomiting, and clouding of consciousness) may root in the increased intracranial pressure or mass effect through invasion, compression, and edema. Symptoms arising from the latter may include seizures, focal neurological deficit, and altered cognitive functions [2], [6]. 

GBM is generally regarded as among the most common primary CNS tumors. Though this neoplasm is currently categorized more often histologically, genetic studies have recently been positioned as an integral part of the diagnosis, disease profiling, and prognostic assessments [2], [10], [15]. 

In practice, the treatment approaches are pursued based on the patient’s age, performance status, and baseline neurocognitive status. In many instances, the standard care comprises surgical resection followed by chemoradiation and adjuvant chemotherapy in cognitively competent patients younger than 70 years old. In elderly subjects however, radiotherapy and palliative care is perhaps all that is sought [28]. 

Despite the best possible care today, GBM prognosis has remained poor given the aggressive nature of the tumor. Therefore it is crucial to ensure that not only an extended progression-free survival but also an acceptable quality of life is provided through treatments. In terminal cases where treatments are withheld, best supportive or palliative care has to be provided to ameliorate symptoms as much as possible [28]. 

## 3 Clinical assessment and follow-up of patients with GBM

The general, neurological and cognitive examination constitute the key imperatives while evaluating a case with GBM. When baseline measures are established, an improvement in the patient’s clinical status can be witnessed, in terms of intracranial hypertension, focal neurological signs or cognitive profile. The assessment needs to be dynamic based on the treatment response [28], [29]. 

As the clinical status improves, evaluation of the patient may advance to more sophisticated tests including neuropsychological evaluations, language assessments, neurocognitive profiling, and rehabilitation or studies where the patient’s cooperation is definitional, including the functional magnetic resonance imaging (fMRI) of functional near infra-red spectroscopy (fNIRS) [30].

The functional status of patients is usually reported by the Karnofsky performance status (KPS) score or Eastern Cooperative Oncology Group (ECOG) scale, where patients with KPS of 70 and above or ECOG score of 0 to 1 have maintained their autonomy and can actively engage in activities of daily living [31], [32]. 

Other than comprehensive neurological examination, patients would need to be assessed for neurocognitive performance and their quality of life. As such pre- and post-treatment quality of life and neurocognitive manifestations would be comparatively measured as a part of clinical evaluations and follow-up [12], [28]. Some validated tools which are used for neurocognitive assessment include testing batteries such as Addendrook’s Neurocognitive Examination (ACE), Repetitive Battery for the Assessment of Neuropsychological Status (RBANS), the Trail Making Test and the Multilingual Aphasia Examination [30]. 

Given the availability of the neurocognitive assessment and rehabilitation platform in our setting [30], the NOSC panel agreed that such evaluations (baseline, post-operatively, and following treatments) using neuropsychological, cognitive tests and functional neuroimaging modalities should be incorporated into our glioma treatment workflow [33]. 

## 4 Treatment protocols and approaches

The choice of treatment is essentially determined by the patient’s age, general medical condition, KPS, characteristics of the lesion, extent of surgical removal, survival benefits against risks and patient’s willingness [28].

The open surgery, navigation-guided or stereotactic biopsy taking can be done to provide histopathological sample to confirm diagnosis. The selection of the biopsy site is crucial since taking samples from necrotic, edematous areas and areas near the subarachnoid space with the potential of bleeding may be misleading or life-threatening. A multidisciplinary neuro-oncology group may best decide on the most appropriate site to be biopsied [5].

Moreover, the surgical resection of the lesion is done to help decompressing the cerebral tissue, especially when the compressive effects threats patient’s life or function. Compared to biopsy only, surgery is known to improve the patient’s condition and to contribute to extended survival [5], [8]. The improved condition of patients following surgery may enable their access to further treatment measures and the opportunity to respond [8].

To minimize the post-surgical sequellae, functional surgery and awake craniotomy settings have been employed in some centers across our country. To maximize the functional outcome, proximity to eloquent areas (motor, language, sensory, visual), baseline focal neurologic signs and deep location of the tumor (basal ganglia, ventricles, brainstem) need to be well considered upon surgery [2], [6].

Baseline neuropsychological assessments and brain mapping measures for surgical planning would provide useful baseline information. Furthermore, stereotactic radiosurgery or safe maximal resection will be done, the latter including intraoperative recording through electrocorticography or somatosensory evoked potentials where applicable. The setup has already been established in well-equipped neuroscience laboratories in Shiraz [30]. 

### 4.1 Primary and complementary treatments in GBM

The choice of treatment in patients younger than 70 years with a KPS of 70 and above is based on the latest EANO guideline [28]. Maximal surgical excision is in fact the practice at first place. Although some years ago, the prognostic impact of surgery was questioned, different studies confirm that in high grade gliomas, extensive surgery is linked to a more favorable prognosis [34], [35], [36], [37], [38].

The standard treatment in GBM is based on the results from an international, multi-center open-labeled, randomized controlled phase III study, where patients who were randomized to undergo radiotherapy (RT) alone or concurrent chemoradiation with temozolomide (TMZ) at 75 mg/m^2^ followed by 6 cycles of TMZ at 150–200 mg/m^2^ where compared in terms of overall survival (OS) [21].

According to Stupp’s study, a larger two-year OS rate was observed in cases receiving RT+TMZ than RT alone (26% vs. 10%). In a follow-up investigation on the same study population, 9% of GBM patients who followed this treatment survived up to 5 years, while this was only 1% in the RT alone arm. Additionally, the impact on OS was superior in patients with positive methylation status in the O6-methylguanine-DNA-methyltransferase (MGMT) promoter [21].

More recently, further possible treatments targeting to arrest angiogenesis have also been experienced in clinical setting. For instance, in a multi-center, international, randomized phase III trial known as AVAglio, an anti-angiogenic therapy provided survival benefits in newly diagnosed GBM patients [39], [40]. This study randomly assigned patients into two arms whereby the first underwent the standard protocol by Stupp et al. plus placebo, while the second followed the similar standard treatment plus a concurrent dose of bevacizumab at 10 mg/kg i.v., the treatment continuing for 3 additional weeks. Results of the above investigation indicated an improved progression-free survival (PFS) and quality of life in the concurrent treatment arm. This could have largely been due to a reduced use of steroids secondary to the add-on therapy using bevacizumab. This study however failed to demonstrate improvement in OS [39].

In the current practice of neuro-oncology, bevacizumab is particularly indicated in recurrent GBM settings. The regimen is normally administered at 10 mg/kg i.v. biweekly, or 15 mg/kg i.v. every 3 weeks [12], [28]. This approach is being followed in our practice given the availability of bevacizumab.

Other available alternative chemotherapeutic regimen to consider include the procarbazine, lomustine and vincristine (PCV) combination protocol [28]. 

In the recurrent GBM setting, some challenging cases who fail to respond to the above, may be enrolled in investigational setups including immunotherapy (PD-1, PD-L1 or CTLA-4 receptor antibodies) [41], [42]. 

### 4.2 Treatment follow-up

In controversial cases, an interdisciplinary neuro-oncological assessment would help determining the most advisable therapeutic and follow-up approaches. 

In fact, a crucial component in the patients’ treatment is proper follow-up through interval clinical, imaging and laboratory assessments. The follow-up is normally pursued with the post-operative control MRI to define the extent of residual lesion following surgery. In addition, patients undergo multimodal imaging using MRI to assess their tumor behavior as well as the response to treatment [4], [6].

Moreover, on-treatment laboratory assessments also become applicable to evaluate patients clinically for possible chemotherapy-induced side effects prior to each treatment cycle (typically, every 4 weeks) [28].

In case of uncertainties with regard to tumor behavior in regular MRI, multimodal MRI scans comprising diffusion and perfusion sequences with apparent diffusion capacity (ADC) maps would provide additional information on the extent of the lesion and regional cerebral blood volume (rCBV). To elucidate whether true or pseudo-progression has occurred, uncertain areas might be evaluated using the magnetic resonance spectroscopy (MRS). Such imaging assessments would clarify contrast enhancement or hyper-intensity signals in T1 and T2 sequences, respectively. Other than the above and when accessible, [18F]-fluoro-ethyl-L-tyrosine PET would be the best evaluated option to explore the biological activity of the lesion [12], [28], [43]. 

### 4.3.Control MRI schedule

Post-operatively, the first MR scan is recommended at 48 hours. Further to regular follow-up imaging upon treatment, a diffusion-weighted MRI might be required to define the presence of any subacute contrast enhancement which might be mistaken for progression while in fact resulting from radiation necrosis in typical instances [12].

While the need for control MRI upon completion of radiotherapy treatment is a matter of debate, the first control MRI is strongly advised following the 2^nd^ or 3^rd^ cycle of adjuvant chemotherapy. Control MRIs would then be taken every 8–12 weeks to evaluate the patient’s responses to therapy [12], [28]. 

### 4.4 Neuroimaging technical characteristics and considerations 

The control and follow-up MRI need to be practiced using the same equipment, acquisition protocol, contrast and topographical references, to technically allow comparable data [44].

The sequences applied to follow-up MR scans include the T1 3D and gadolinium-contrasted T1 3D spoiled gradient recalled (SPGR) images providing three-plane reconstruction and size calculation through segmentation as well as T2 or T2 fluid-attenuated inversion recovery (FLAIR) with thin cuts [44].

In practice, CT scans have no place in follow-up assessment of patients with CNS tumors unless complications including hydrocephalus or hemorrhage are doubted [13]. 

### 4.5 Response assessment in neuro-oncology (RANO) criteria

The RANO criteria, based on the MRI scans, are currently referenced for response assessment in neuro-oncology. The radiological response to a given therapy is evaluated after measuring baseline tumoral dimensions before and after the treatment. It should always be noted that radiological response assessment particularly upon permeability changes after treatment with anti-angiogenic therapies is challenging. Thus, confirmatory imaging needs to be considered 4 weeks following a radiological response [45], [46].

With respect to the radiological response, key terms including complete response (CR), partial response (PR), stable disease (SD), and progressive disease are applied. Disease progression is usually considered when T2/FLAIR non-enhancing lesions are increased in size on stable or increased corticosteroid doses compared to baseline or earlier assessments [46]. 

When following up the disease, the possibility of pseudo-progression needs to be taken into account. In other words, early tissue reactions within the first few months should be differentiated from similar effects which in fact result from true progression. Some factors to consider when labeling true progression include:

1. contrast enhancement beyond 12 weeks after the completion of radiotherapy,
2. contrast enhancement in a non-irradiated zone,
3. an increase in non-enhancing and hyper-intense signals on T2/FLAIR,
4. excluded underlying causes (ischemia, post-radiotherapy changes, seizure activity, infection or demyelination) explaining the changing T2/FLAIR signals, and
5. altered T2/FLAIR signals indicating tumor infiltration outside the radiation field [46].

## 5 The NOSC’s agreed clinical pathway in glioma care

**Figure 1:**
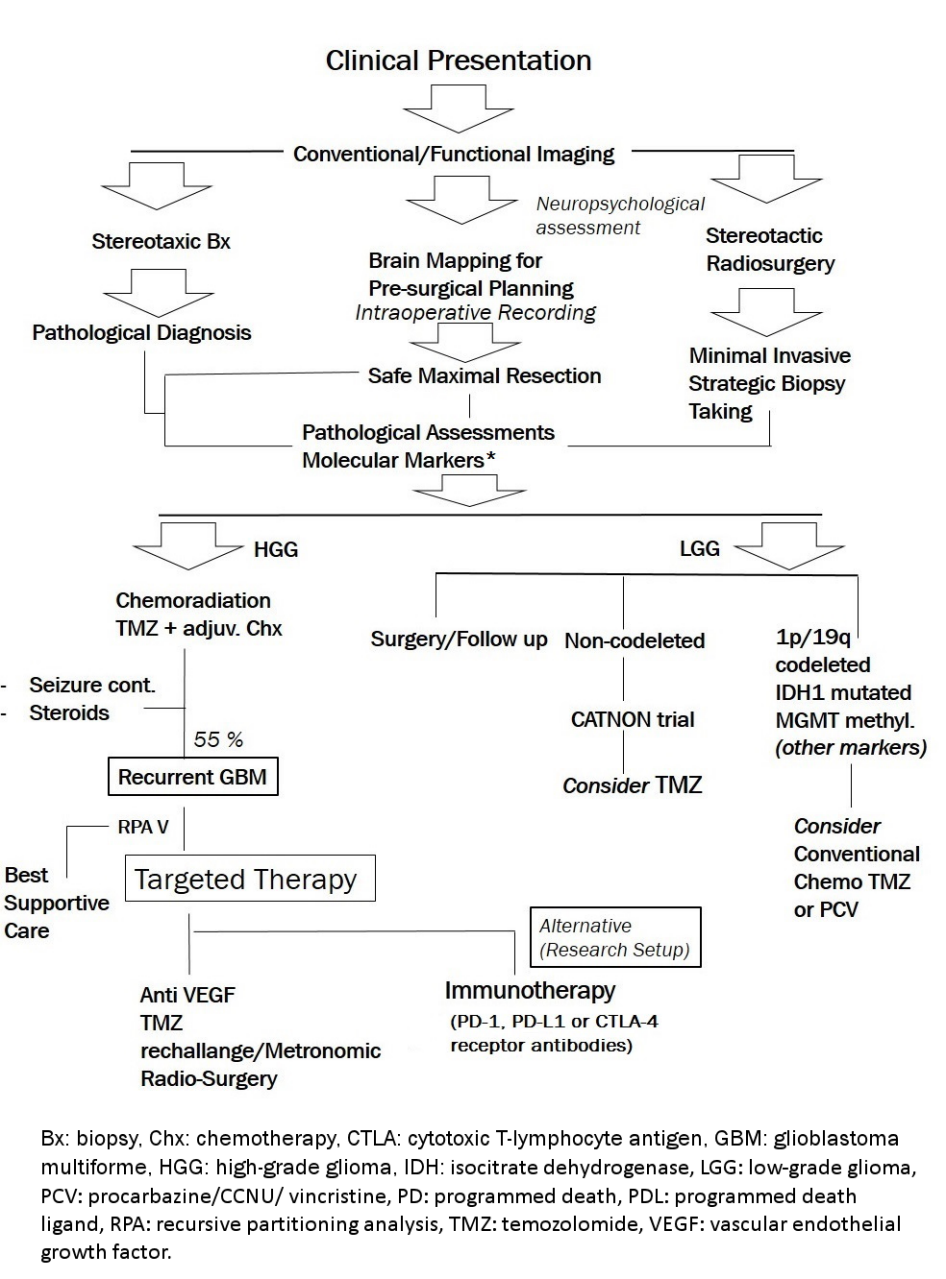
The NOSC’s agreed clinical pathway for the diagnosis and treatment of patients with glioma. Early diagnosis, history, clinical examination, neuroimaging, preoperative management, biopsy and resection, histological classification and grading, molecular diagnostics as well as surgical therapy, radiotherapy, pharmacotherapy, and other therapeutic approaches are considered in the pathway. The pathways would be expected to receive updates in coming years when molecular markers testing set-up and novel therapies become widely accessible in our setting.

Based on the international consensus statements and the review of current guidelines [15], [28], our working group’s agreed approach to patients with malignant gliomas, including diagnostic aspects (i.e., early diagnosis, history, clinical examination, neuroimaging, preoperative management, biopsy and resection, histological classification and grading, molecular diagnostics) as well as general therapeutic approach (i.e., surgical therapy, radiotherapy, pharmacotherapy, and other therapeutic approaches) is depicted in Figure 1 (Fig. 1). 

The routine use of molecular markers (PD-1, PD-L1 or CTLA-4 receptor antibodies) especially for the classification purpose, as recommended by 2016 WHO guideline, is currently not feasible and yet to be established in our setting. 

## 6 Summary and recommendations

In line with the mission statement of the NOSC in Iran, and in order to draw an updated clinical decision guide for the interdisciplinary practice of neuro-oncology, and the treatment of high-grade gliomas in particular, the panel of experts from Shiraz NOSC agreed on a clinical pathway (Figure 1 (Fig. 1)). Based on this clinical pathway algorithm following the clinical suspicion, conventional/functional neuroimaging are taken, after which stereotactic biopsy would be taken to ensure pathological diagnosis. The specimen is then assessed for molecular markers. Patients will initially undergo baseline neuropsychological assessments and further brain mapping measures for surgical planning. Stereotactic radiosurgery or safe maximal resection will be done, latter including intraoperative recording through electrocorticography or somatosensory evoked potentials where applicable. 

Post-operatively, the newly diagnosed HGG patients would undergo the protocol of chemo-radiation and adjuvant chemotherapy with TMZ established by Stupp et al. [12]. Seizure control and optimized steroid use are to be considered. 

In recurrent HGG patients, those with poor performance status receive best supportive care, whereas patients with fair to favorable performance either receive targeted/re-challenge therapy (anti-angiogenic agent, TMZ re-challenge/metronomic dosing, radiosurgery, re-irradiation or TMZ+anti-angiogenic agent) or enter the investigational setups including immunotherapy. 

Providing an optimal care to patients with CNS malignancies requires a multidisciplinary team approach. The key benefits include well-organized coordination of multiple providers, direction for complicated cases, open communication amongst care teams, education, and clinical trial access. Over the past 6 years, the NOSC panel has strived to coordinate multidisciplinary care and influence care decisions in most of the major provinces across Iran.

The following parts of the international consensus statements [15], [28] have been established by the NOSC panel for implementation in our local practice:

1. The standard of care for anaplastic astrocytoma includes resection as feasible or biopsy, followed by involved field radiotherapy.
2. The standard of care for glioblastoma (age <65–70 years) includes resection as feasible or biopsy, followed by involved-field radiotherapy and concomitant and adjuvant (six cycles) TMZ chemotherapy.
3. Chemotherapy with TMZ or PCV is as effective as radiotherapy in the treatment of anaplastic gliomas, including anaplastic astrocytomas.
4. Elderly patients who are not candidates for TMZ concurrent with radiotherapy followed by adjuvant TMZ should be treated with radiotherapy (e.g., 15 doses of 2·66 Gy) alone or temozolomide (5/28) based on MGMT promoter methylation status
5. Upon recurrence, standards of care for GBM are nitrosourea regimens, TMZ rechallenge, and bevacizumab as options for pharmacotherapy.
6. Patients with 1p/19q co-deleted anaplastic oligodendroglial tumors (assessed in our country through FISH method in major provinces) should not be treated with radiotherapy alone, but should receive chemotherapy with alkylating agents with or without radiotherapy.

Meanwhile, some remaining parts of the international consensus, especially the recent WHO molecular classification for gliomas, are planned to be established in the coming years when almost all our CNS malignancy care accounts are equipped with such molecular testing set-ups (1p/19q co-deletion, MGMT promoter methylation, IDH1/2 mutation).

While there is strong agreement on the role of clinical decision making and education, the countrywide implementation of recommendations need to be continuously monitored and ensured by the NOSC provincial founding panels across the country. 

## Notes

### Acknowledgment

The authors would like to thank Dr. Dindoust P, Salarian A, Hejazi-Farahmand, SAR for supporting this clinical forum. The ‘NOSC, Shiraz’ received scientific and administrative support from the Department of Radiation Oncology, Shiraz University of Medical Sciences, Shiraz, Iran, as well as the MSD Medical team at Behstan Darou PJS, Tehran, Iran. 

### Competing interests

The present report outlined the communications and experts’ opinions during the NOSC meet-up 2015, Shiraz, Iran. The authors declare no competing interest upon data review, talk delivery during the meeting, interactive discussions and preparation of the present report. MN provided medical consultancy to Behestan Medical Scientific Committee, Behestan Group, Tehran, Iran.

### Preprint publication

A first version of this manuscript was published as a not peer-reviewed draft in the PeerJ Preprints [47].
